# SEOSS-Queries - a software engineering dataset for text-to-SQL and question answering tasks

**DOI:** 10.1016/j.dib.2022.108211

**Published:** 2022-04-27

**Authors:** Mihaela Todorova Tomova, Martin Hofmann, Patrick Mäder

**Affiliations:** a Technische Universität Ilmenau, Ilmenau 98693, Germany; b Faculty of Biological Sciences, Friedrich Schiller University, Jena 07745, Germany

**Keywords:** Software and systems requirement engineering, Text-to-SQL, Dataset, Question answering, Natural language processing

## Abstract

Stakeholders of software development projects have various information needs for making rational decisions during their daily work. Satisfying these needs requires substantial knowledge of where and how the relevant information is stored and consumes valuable time that is often not available. Easing the need for this knowledge is an ideal text-to-SQL benchmark problem, a field where public datasets are scarce and needed. We propose the SEOSS-Queries dataset consisting of natural language utterances and accompanying SQL queries extracted from previous studies, software projects, issue tracking tools, and through expert surveys to cover a large variety of information need perspectives. Our dataset consists of 1,162 English utterances translating into 166 SQL queries; each query has four precise utterances and three more general ones. Furthermore, the dataset contains 393,086 labeled utterances extracted from issue tracker comments. We provide pre-trained SQLNet and RatSQL baseline models for benchmark comparisons, a replication package facilitating a seamless application, and discuss various other tasks that may be solved and evaluated using the dataset. The whole dataset with paraphrased natural language utterances and SQL queries is hosted at figshare.com/s/75ed49ef01ac2f83b3e2.

## Specifications Table

**Table:** 

Subject	Software Engineering
Specific subject area	Software and systems requirement engineering. Minimization risk in the software development process.
Type of data	Table Text
How data were acquired	Utterances are collected from previous studies, software projects, issue tracking tools, and through expert surveys found in literature. The SQLite database based on which the SQL queries corresponding to the utterances were constructed, was chosen from the SEOSS-dataset. The SQL queries are developed to answer the collected utterances given the database. The labeled questions are mined from comments found in databases part of the SEOSS-dataset.
Data format	Raw
Description of data collection	We were motivated by the scenarios described in the literature we analyzed, and questions asked by stakeholders that we found in these scenarios. We formulated SQL queries for 166 corresponding natural language questions using data from issues tracking systems and version control systems. We paraphrased each of these natural language questions six more times, 3 of which in a specific way and 3 in a non-specific way.
Data source location	Institution: Technical University of Ilmenau City/Town/Region: Ilmenau Country: / Germany
Data accessibility	The data is publicly available under the link provided below. Repository name: Figshare Data identification number: 75ed49ef01ac2f83b3e2 Direct URL to data: https://figshare.com/s/75ed49ef01ac2f83b3e2

## Value of the Data

• With the SEOSS-Queries orchestrated dataset we offer machine learning scientists and researchers a dataset specifically designed to train and evaluate text-to-SQL models on data derived from the software engineering domain. Additionally, we provide a large set of questions (SEOSS-Queries issue comments) extracted from issue comments, labeled by issue type that can be of use to researchers who want to understand better software developers’ needs.
• Stakeholders (e.g. developers) of system and software engineering projects can use text-to-SQL models trained on the SEOSS-Queries orchestrated dataset to query-database information faster and easier to perform a task or to make a decision. Machine learning scientists and researchers can use the SEOSS-Queries issue comments dataset to gain further insight into the information needs of software developers by using machine learning and NLP techniques.
• The data from our datasets can be used in the field of Machine Learning and NLP. The SEOSS-Queries orchestrated dataset can be used to train and evaluate text-to-SQL models. The opposite task (SQL-to-text) can be considered in the context of database engineering. The questions from the SEOSS-Queries issue comments dataset can be used for classification and clustering of written user inquiries. Experiments can give insight into text similarities and give hints leading to better prioritization of user inquiries. The data can also be used to further analyze developers’ information needs.

## 1 Data Description

SEOSS-Queries consists of two parts (cp. Table 1).

**Table 1:** Overview of the Spider and our proposed SEOSS-Queries text-to-SQL datasets.

Dataset	# utterances	# classes	# queries	# DBs	# domains	# tables per DB	# ORDER BY	# GROUP BY	# nested queries	# HAVING
Spider [2]										
– full	10,181	–	5,693	200	138	5.1	1,335	1,491	844	388
– SW problems	49	–	24	1	1	6	12	8	12	0

SEOSS-Queries (ours)										
– orchestrated	1,162	3	166	1	1	13	16	49	15	8
– issue comments	393,086	4	–	–	–	–	–	–	–	–

**SEOSS-Queries orchestrated** is a set of natural language utterances available in seven paraphrased versions accompanied by one SQL query each. In total, 166 questions (utterances) were orchestrated. The natural language utterances were primarily derived from literature and were refined to correspond to data found in an issue tracking system (ITS) and version control system (VCS) of a real-world software project (i.e., Apache Pig) that was extracted and persisted into an SQLite-database by Rath et al. [1]. To check if the constructed SQL queries were syntactically correct, they were executed against the database. Based on the literature we examined, we decided to split our utterances manually into two categories: development and research. We marked utterances as ‘development’ that were motivated by papers addressing software needs of stakeholders (e.g., developers, data scientists, etc.) or were derived from typical usage scenarios’ questions of issue tracking systems. Utterances motivated by papers in which data from software repositories such as issue tracking systems or version control systems were used for further analysis were marked as ‘research’. As a result, 81 are categorized as questions to be likely asked in the domain development, and 63 to be likely asked in the domain research. The remaining 22 were motivated by the content in questions stakeholders asked within the comments section of issues of type bug, enhancement/improvement, new feature/feature request, and tasks of 33 open-source Apache projects extracted and persisted into databases by Rath and Mäder [1]. In the supplementary materials in Table 1, we list the 166 utterances, their category, and the source from which they were motivated.

**SEOSS-Queries issue comments** is a set of labeled questions derived from issue comments discussions extracted from 33 prominent Apache open-source software projects. After analyzing the issue types in all 33 projects, we decided to look at comments of issues that are of the following four types: bug, enhancement/improvement, new feature/feature request, and tasks, since they were present in almost all the projects.^1^ In total, we extracted 393,086 questions from comments of types bug, enhancement/improvement, new feature/feature request, and tasks. We analyzed some of the questions in the comments more precisely and based on their content derived part of the utterances in the SEOSS-Queries orchestrated dataset. Furthermore, the content derived from questions found in issue comments contains valuable information about how developers communicate with each other, how they express themselves, and what information needs they have. From a semantic and syntactic point of view, these questions can interest the NLP community. Understanding stakeholders’ information needs in the software engineering domain is an undergoing research. Analyzing developers’ questions from real-world software projects can thus further support this research. In a CSV-file, provided in our Figshare repository, we listed each question, the project it belongs to, and the issue type of the comment from which the question was extracted.

Table 1 gives an overview of Spider (a popular large text-to-SQL dataset) and SEOSS-Queries (our dataset). Table 2 gives an example of natural language utterances from the SEOSS-Queries orchestrated that were incompatible with the baseline models we chose. To check the validity of the SEOSS-Queries orchestrated dataset we perfomed 4 experiments, which results we present in Tables 3, 4, 5, and 6.

**Table 2:** Examples of utterances and SQL queries that are incompatible to the Spider grammar.

Problematic SQL query or NL utterance	Incompatibility
List the descriptions and attachments’ files names that belong to the issue with id PIG-3599	quote in question
SELECT CASE WHEN EXISTS (SELECT * FROM change_set_link WHERE issue_id = ‘PIG-4092’ AND commit_hash = ‘[...]’) THEN ‘True’ ELSE ‘False’ END	CASE statement
SELECT * FROM issue WHERE resolution = “Cannot Reproduce” OR resolution = “Won’t fix”	quote in query
SELECT issue_id FROM issue WHERE strftime(’%Y-%m-%d’, created_date) = DATE(’now’,’-1 day’)	srftime() function
SELECT * FROM issue AS T1 WHERE T1.issue_id NOT IN (SELECT T2.issue_id FROM issue_attachment AS T2)	NOT in WHERE clause
SELECT * FROM issue WHERE type = ‘Bug’ and (status = ‘Resolved’ OR status = ‘Closed’) AND (resolution= ‘Fixed’ or resolution = ‘Done’)	brackets in WHERE clauses
SELECT T1.* FROM issue AS T1 JOIN issue_fix_version AS T2 ON T1.issue_id = T2.issue_id WHERE T2.fix_version = ‘0.12.1’	T1.* in SELECT
SELECT AVG(count_per_issue) FROM (SELECT Count(DISTINCT username) AS count_per_issue FROM issue_comment GROUP BY issue_id)	keyword AS in SELECT
SELECT Count(*) FROM issue WHERE description IS NULL	NULL in WHERE clause

**Table 3:** Results of experiment 1: SEOSS-Queries evaluation with models pre-trained on Spider.

	easy	medium	hard	extra hard	all
count	392	378	77	84	931
**exact match accuracy (SQLNet)**	0.023	0.000	0.000	0.000	0.010
**exact match accuracy (RatSQL, Glove)**	0.309	0.214	0.091	0.000	**0.224**
**exact match accuracy (RatSQL, Bert)**	0.161	0.201	0.065	0.012	0.156

**Table 4:** Results of experiment 2: all queries trained and left-out utterances used for evaluation.

	easy	medium	hard	extra hard	all
count	112	108	22	24	266
**exact match accuracy (RatSQL, Glove)**	0.911	0.806	0.636	0.292	**0.789**
**exact match accuracy (RatSQL, Bert)**	0.768	0.648	0.364	0.250	0.639

**Table 5:** Results of experiment 3: evaluation with 20% untrained queries.

	easy	medium	hard	extra hard	all
	**all utterances**				
count	35	98	21	35	189
**exact match accuracy (RatSQL, Glove)**	0.743	0.357	0.619	0.143	**0.418**
**exact match accuracy (RatSQL, Bert)**	0.743	0.337	0.143	0.114	0.349

	**only non**-**specific utterances**				
count	15	42	9	15	81

**exact match accuracy (RatSQL, Glove)**	0.533	0.190	0.667	0.067	**0.284**
**exact match accuracy (RatSQL, Bert)**	0.533	0.143	0.222	0.000	0.198

	**only specific utterances**				
count	20	56	12	20	108

**exact match accuracy (RatSQL, Glove)**	0.900	0.482	0.583	0.200	**0.519**
**exact match accuracy (RatSQL, Bert)**	0.900	0.482	0.083	0.200	0.463

**Table 6:** Results of experiment 4: evaluation with balanced specific and non-specific utterances.

	easy	medium	hard	extra hard	all
	**all utterances**				
count	112	108	22	24	266

**exact match accuracy (RatSQL, Glove)**	0.866	0.806	0.591	0.333	**0.771**
**exact match accuracy (RatSQL, Bert)**	0.732	0.574	0.364	0.083	0.579
	**only non-specific utterances**				
count	56	54	11	12	133

**exact match accuracy (RatSQL, Glove)**	0.839	0.704	0.636	0.250	**0.714**
**exact match accuracy (RatSQL, Bert)**	0.607	0.389	0.364	0.000	0.444

	**only specific utterances**				
count	56	54	11	12	133

**exact match accuracy (RatSQL, Glove)**	0.893	0.907	0.545	0.417	**0.827**
**exact match accuracy (RatSQL, Bert)**	0.857	0.759	0.364	0.167	0.714

**Fig. 1:**
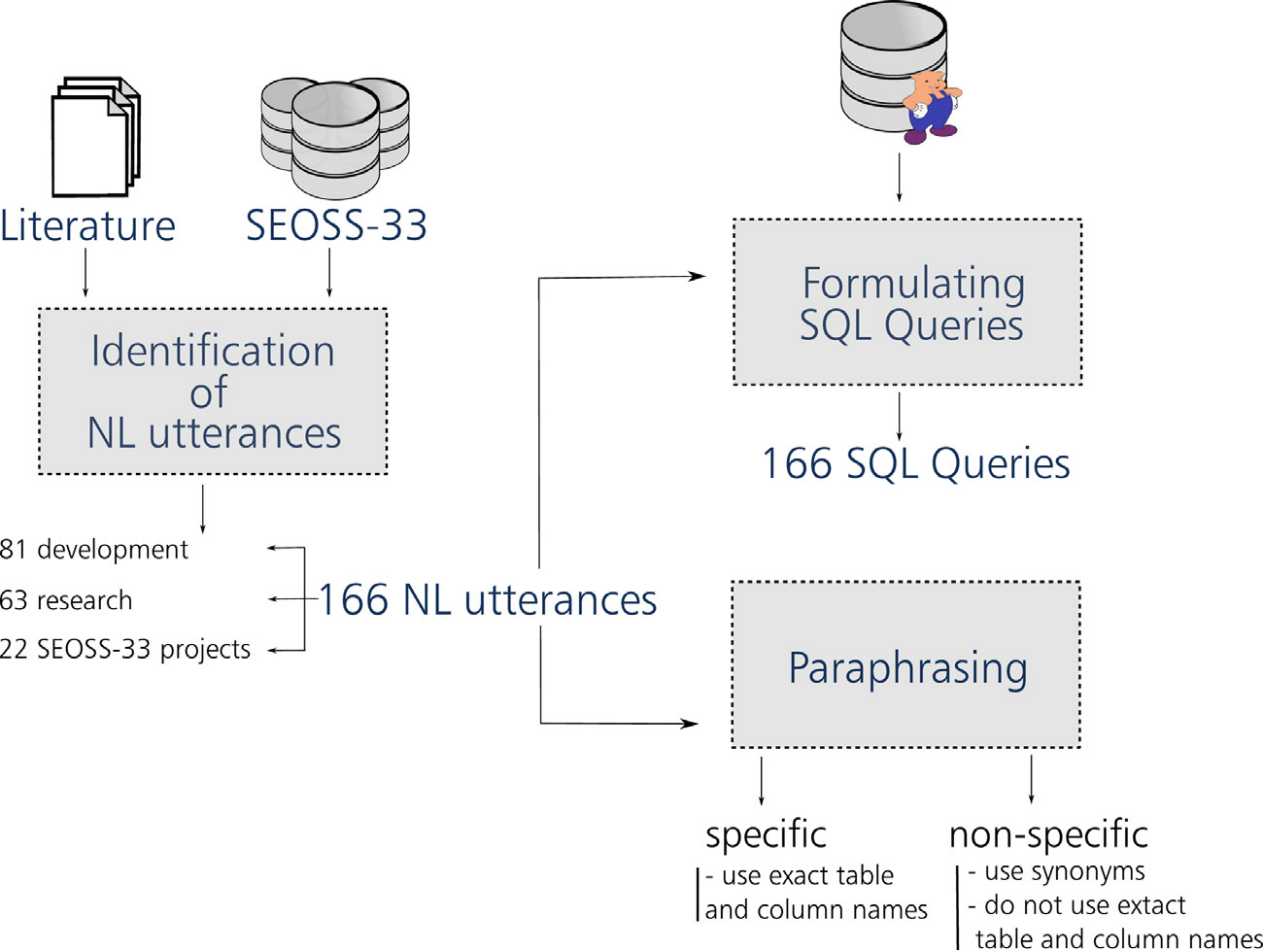
Main steps of constructing SEOSS-Queries orchestrated.

**Fig. 2:**
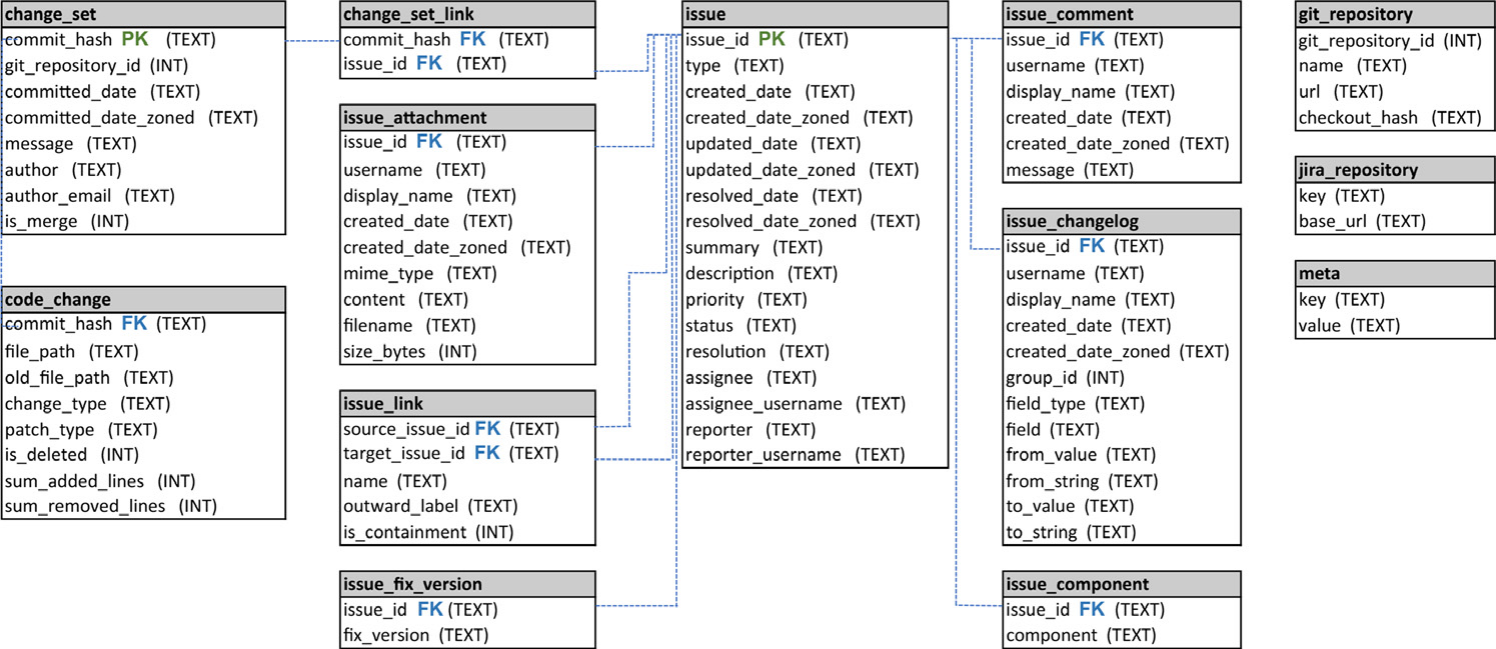
Database schema of the Pig database.

**Fig. 3:**
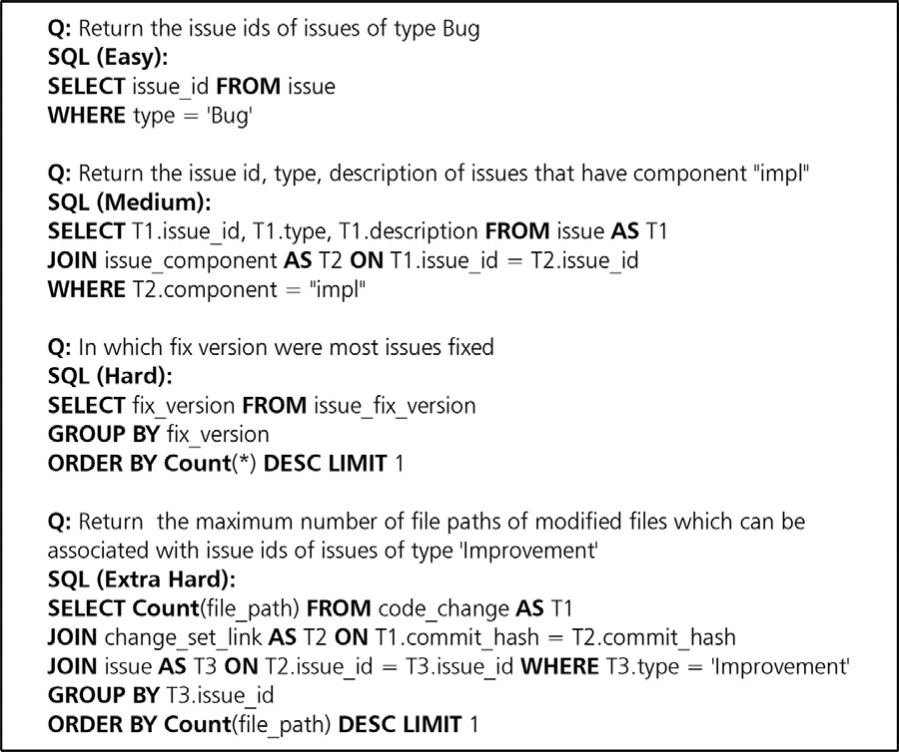
Examples of different hardness levels of SQL queries from the SEOSS-Queries Dataset.

Fig. 1 depicts the main steps used to construct the SEOSS-Queries orchestrated dataset. Fig. 2 depicts the database schema of the Apache Pig project, used as a way to check the correctness of the formulated SQL queries in our dataset. Fig. 3 gives an example of the different hardness levels of SQL queries from our dataset. Fig. 5 depicts the main steps used to construct the SEOSS-Queries issue comments dataset.

As supplementary materials in our Figshare repository, we provide the two datasets described in this manuscript as CSV files (seoss_queries_orchestrated and seoss_queries_issue_comments). Furthermore, we provide steps and data files needed to reproduce the experiments with which we checked the validity of our SEOSS-Queries orchestrated dataset. In the “read.me” file, we provide all necessary steps to execute the experiments. We included the database (apache-pig.sqlite) and database schema (schema.sql) of the Apache Pig project based on which the SQL queries were formulated and against which their correctness was ensured. The approaches we chose to perform the experiments to test the validity of our dataset could not handle all of the utterance-SQL pairs in the SEOSS-Queries orchestrated dataset. We thus provide an additional CSV file (seoss_queries_used_for_experiments) with all utterance-SQL pairs that were used to test the validity of our dataset. In the folder “reproduction”, we include files needed to train and evaluate the baseline models we used to validate our dataset. Details of each experiment are included in separate folders titled “experiment_x”, x represents the experiment number. We included the files needed to perform the training and evaluation of the baseline models we chose in each experiment folder. The file “tables.json” gives details of all database tables used during training and evaluation of a model. Originally, “train_others.json”^2^ includes only training data from the Spider dataset. We, however, modify this file by appending data from our dataset in it. Files named “dev.json” were also modified with data from our dataset that needs to be evaluated by the model of our choice. To evaluate data only from our dataset, we included the predicted results (predicted_x_x_x.txt) of each experiment and ground-truth results (gold_x_x.txt), as well as the tables information (tables_pig.json) of the database we used for the evaluation.

## 2 Experimental Design, Materials and Methods

### 2.1 SEOSS-Queries orchestracted

In Fig. 1 we depict the main steps to construct the SEOSS-Queries orchestrated dataset.

**Identification of natural language utterances.** We orchestrated natural language utterances into SEOSS-Queries that are likely to be asked by developers or researchers. To do this we used snowball sampling to find literature in which the day-to-day information needs of software developers was studied or in which interviews were presented that determined developers’ specific questions as sources for the natural language utterances that they likely ask. We found developers to need often information, like “who is working on what,” “who solved what,” “what was changed,” “what files are related to a change,” as well as information from a specific time.

Based on the questions we found and based on the content of the SEOSS-33 projects, we refined the respective questions. Furthermore, we also included typical usage scenarios’ questions of issue tracking systems3,4,^5^. We refer as well to studies that analyze issue tracking systems and code repositories to understand and support development processes. Some cases contain formulated questions as sources for natural language utterances that are likely to be asked by researchers [3], [4], [5], [6], [7], [8], [9], [10], [11]. Researchers are interested in more quantitative information (e.g., “How many issues are not linked to change sets?”, “Count all distinct issue types.”, “Count issues of type Bug.”), while developers request more specific information to solve a problem or to make decisions (e.g., “Find all issues which either have the status ‘closed,’ ‘resolved’ or ‘patch available.”’, “Who is the assignee working on an issue with id PIG-3599?”, “What is the issue type of issue id PIG-3599?”). Researchers’ information needs often refer to a project’s characteristics, such as the number of bugs or improvements in a project [9] or the number of related issues [5], or they assess quality measures (e.g., “rate of fixed issues of type bug”) [3].

In addition to the natural language utterances strictly derived from literature, we identified utterances from questions extracted from 1,440,941 issue comments of issue type bug, enhancement/improvement, new feature/feature request, and tasks that are part of the 33 projects in the SEOSS dataset provided by Rath and Mäder [1]. Given the significant number of questions we extracted we decided first to narrow them down. Thus, we looked only at questions that contained column names from the database schema of our project. Based on their content, we extended our dataset with natural language utterances that can be considered a resulting action from the discussions in the questions. For example, developers discuss changing the priority or resolution of an issue, or they want to contact other developers by email to exchange information or ask for help. To do this, one must first find out what priorities or resolutions are specified in the project or the email addresses of developers working in it. Hence, one must first list them.

In total, we orchestrated 166 questions (utterances), 81 are categorized as questions to be likely asked in the domain development, and 63 to be likely asked in the domain research. The remaining 22 were motivated by the content in questions stakeholders asked within the comments section of issues of type bug, enhancement/improvement, new feature/feature request, and tasks. We precisely formulated the orchestrated natural language utterances to contain the exact column- and table-names from the database, minimizing different interpretations as much as possible. Table 1 from the supplementary materials shows all 166 of these questions.

**Selecting and adapting a development project.** We used content from ITSs and VCSs extracted and persisted into an SQLite-database by Rath et al. [4]. Each of the 33 databases contains the complete information of the respective project’s issue tracking system and the code version repository at the time of collection. For this manuscript, we chose the Pig project to perform baseline experiments with which we evaluate the applicability and the validity of our dataset. Apache Pig^6^ is “a platform for analyzing large datasets that consists of a high-level language for expressing data analysis programs, coupled with infrastructure for evaluating these programs” and seems to be a representative choice being a long-term developed and maintained mid-size open-source project. Fig. 2 shows the schema of the Pig database. The database schema reflexes the schema of all 33 SEOSS projects.

**Formulating the SQL queries.** For each of the 166 natural language utterances an SQL query was formulated. Cases in which more than one possible SQL queries were formulated, were discussed and at the end only one was picked. Each SQL query was executed against the Apache Pig database to ensure the correctness of the formulated SQL query.

**Paraphrasing the natural language utterances.** To provide diversity to the dataset, we decided to formulate paraphrased versions of the 166 natural language utterances that we have collected so far. The paraphrased utterances were not machine-generated since we wanted to represent how an individual would formulate questions.

Given the initial 166 natural language utterances, we aimed to create specific and non-specific paraphrased versions to account for the variety of natural language. We ended up formulating six paraphrased natural language utterances. Three of which are paraphrased in a **specific** way, including the relevant column names and table names appearing in the corresponding SQL query, and three in a **non-specific**, aiming for a less precise formulation.

In both versions, we varied words that are used frequently in natural language utterances such as Return, “List,” “what,” “who.” In the non-specific case, the main rules we tried following were to use synonyms (e.g., “unique” = “distinct” = “different”) or to express things in a different manner (e.g., “between 2014-10-01 and 2014-10-31” = “in the month of October”, “between January and April 2015” = “in the first quarter of 2015”, “created an issue” = “reported an issue”). We as well-formulated examples of syntactically incorrect sentences that did not include a verb (e.g., Any issue ids with a created date between ‘2017-01-01’ and ‘2017-03-31’, Any bugs, Any critical or blocking issues). Furthermore, in cases in which specific words can be linked easily to a column or a table, we tried to express them differently. For example, words like “assigned,” “reported” or “committed” can be easily linked to columns “assignee,” “reporter,” or “committed_date.” In such cases, we used different ways to express the same phrase, e.g., **assigned** → “Who is responsible for,” “Who is working on.”

**Creating baselines for SEOSS-Queries orchestrated.** We chose two main state-of-the-art text-to-SQL methods to generate baselines for our dataset: SQLNet and RatSQL. RatSQL is a top-performing method in the Spider datasets’ leaderboard^7^, and SQLNet is a frequently employed baseline method for text-to-SQL approaches.

**Fig. 4:**
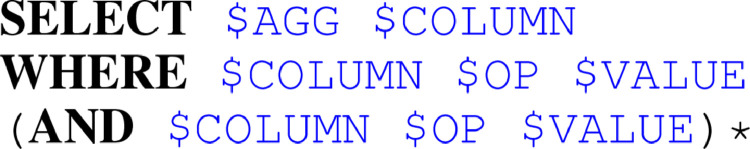
SQLNet-sketch, adapted from Xu et al. [12].

SQLNet by Xu et al. [12] is a sketch-based approach. In Fig. 4 we present the sketch used by SQLNet. Tokens beginning with $ represent slots that can be aggregation operators, column, value, or one of the following symbols: >,<,=. In sketch-based approaches it is only necessary to predict the content in an SQL query in the form of slots without the need of predicting the SQL grammar of the query [13]. In their work Xu et al. propose separate models for the generation of slot content in a SELECT clause and WHERE clause of an SQL query, making use of two main techniques: sequence-to-set and column attention. Sequence-to-set is used to predict which column names appear in a subset of interest by computing probabilities given a column name and a natural language utterance. Column attention is used to capture dependency relationships defined in the sketch during prediction. Initially, SQLNet was trained on the WikiSQL dataset. In our case, however, we used a version adapted to and trained on the Spider dataset.^8^ Wang et al. [14] proposes RatSQL a relation-aware self-attention technique to handle schema encoding, schema linking, and feature representation. Following the original publication, we evaluate RatSQL with a pre-trained Glove embedding as well as a pre-trained BERT embedding. We use SQLNet and RatSQL for the first experiment and then solely RatSQL for all remaining experiments since SQLNet can only handle simple SQL queries referring to one table.

**Fig. 5:**
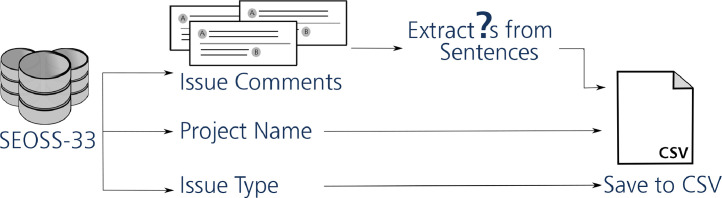
Main step of constructing SEOSS-Queries issue comments.

**Data preprocessing.** We had to ensure that our dataset was compatible with SQLNet and RatSQL initially designed for the Spider dataset. By performing the preprocessing step of the RatSQL approach, we filtered out utterance–SQL pairs from our dataset that were not compatible.

Table 2 shows examples of the 33 natural language utterances and SQL queries that could not be processed and mentions the reason. As a result, we evaluated SQLNet and RatSQL with the remaining 133 utterace-SQL pairs from our dataset. A CSV file listing the remaining 133 utterance-SQL pairs is provided in our Figshare repository as part of the supplementary materials of the paper.

**Categorizing the hardness level of the SQL queries of SEOSS-Queries orchestrated.** Text-to-SQL models may perform differently given how complex an SQL query is, which can be of use during the evaluation of the performance of a text-to-SQL model.

To give an idea of the “hardness” level of the SQL queries in our dataset we used the evaluation script of Yu et al. [2] to categorize our SQL queries into four levels of hardness: easy: 56, medium: 54, hard: 11, extra hard: 12. Examples of each hardness level can be found in Fig. 3. Due to the evaluation scripts’ grammar limitations, the remaining 33 could not be processed.

**Evaluation metrics.** We used exact match accuracy as an evaluation metric across all experiments. Exact match accuracy is the official evaluation metric of the Spider dataset [15] and it measures the equality of the gold (i.e. the ground truth) and predicted SQL query. The exact match accuracy handles the “ordering issue” [12] (e.g. (resolution = ‘Fixed’ or resolution = ‘Done’) = (resolution = ‘Done’ or resolution = ‘Fixed’)) by decomposing the SQL components of the gold and predicted queries into bags of several sub-components and then matching them [2].

Furthermore, we used exact match accuracy based on difficulty level considering four difficulty levels: “easy,” “medium,” “hard,” and “extra hard”. Thereby, the difficulty level is determined based on the type and number of SQL concepts contained in a SQL query [2]. SQL queries marked as “extra hard” can contain, e.g., multiple JOINS, or nested queries, whereas SQL queries marked as “easy” are more straightforward and may not contain even a WHERE clause. The results based on difficulty level were of interest to us since we can use them to better understand what SQL queries the model can handle.

**Checking the validity of our dataset.** We decided to perform four experiments to show the validity of our dataset regarding data quality and quantity. For each of the experiments, we used the evaluation script provided by Yu et al. [2] to compute the exact match accuracy as well as the Spider dataset distributed under the CC BY-SA 4.0 license.^9^ Depending on our experiments, we appended parts of our dataset to Spider’s train (i.e. train_others.json) and dev sets (dev.json). The training and evaluation in all four experiments were performed based on the steps provided in the GitHub repositories of SQLNet^10^ and RatSQL^11^ of the approaches we chose.

In all four experiments, the inputs to the models were a natural language utterance and information about the database schema. Both were stored in JSON-files. The output was an SQL query. For the **first experiment**, we trained all models on Spider and evaluated our dataset. For the **second experiment** we extend the Spider training set with our dataset leaving one specific and one non-specific utterance per query. With the first two experiments, we intended to evaluate the applicability of our dataset.

For the **third experiment**, we extend the Spider training set with a split of 80% of the SQL queries and their respective utterances, leaving 20% never seen queries for the evaluation. For the **fourth experiment**, we extend the Spider training set with two specific and two non-specific utterances per query and use one specific and one non-specific utterance per query for evaluation creating a balanced training in terms of specificity. The last two experiments were created with the idea in mind to evaluate the performance of text-to-SQL models in cases in which the natural language utterances can be either very specific or more general.

Table 3,4,5 and 6 show the experiments results and our dataset's applicability.

The steps necessary to recreate the results from the experiments are provided in the supplementary materials.

### 2.2 SEOSS-Queries issue comments

In Fig. 5 we depict the steps we used to extract questions from issue comments.

We split each comment into sentences to extract questions from comments via the nltk sentence tokenizer. In some cases, the sentences contained a new line; we further filtered them and split them into separate lines. In the end, we checked if each sentence/line ends with a question mark. In a CSV-file we included the question, the project we extracted the question from, and the issue type of the comment from which we extracted the question. With this dataset, one can gain insights into the structure of questions asked in software projects as well as to use the content in them to analyze how developers communicate with each other and what information needs they have.

## Ethics Statement

The dataset does not contain personal or confidential data.

## CRediT authorship contribution statement

**Mihaela Todorova Tomova:** Conceptualization, Methodology, Software, Data curation, Writing – original draft, Writing – review & editing. **Martin Hofmann:** Conceptualization, Methodology, Software, Data curation, Writing – review & editing. **Patrick Mäder:** Conceptualization, Writing – review & editing, Funding acquisition.

## Declaration of Competing Interest

The authors declare that they have no known competing financial interests or personal relationships which have, or could be perceived to have, influenced the work reported in this article.

## Data Availability

Dataset for hierarchical tetramodal-porous architecture of zinc oxide nanoparticles microfluidically synthesized via dual-step nanofabrication (Original data) (Mendeley Data). SEOSS - Queries (Original data) (figshare).

## Appendix A Supplementary materials

Supplementary Data S1 Supplementary Raw Research Data. This is open data under the CC BY license http://creativecommons.org/licenses/by/4.0/ Supplementary Data S1

## Footnotes

1 Project Derby was the only one that did not have issues of type enhancement.

2 https://github.com/taoyds/spider

3 https://www.atlassian.com/blog/jira-software/jql-the-most-flexible-way-to-search-jira-14

4 https://www.atlassian.com/software/jira/guides/expand-jira/jql#visualize-results

5 https://support.atlassian.com/jira-software-cloud/docs/advanced-search-reference-jql-fields/

6 https://pig.apache.org/

7 https://yale-lily.github.io/spider

8 https://github.com/taoyds/spider/tree/master/baselines/sqlnet

9 https://creativecommons.org/licenses/by-sa/4.0/legalcode

10 https://github.com/taoyds/spider/tree/master/baselines/sqlnet

11 https://github.com/microsoft/rat-sql
