# Supplementary material for: SEOSS-Queries - a software engineering dataset for text-to-SQL and question answering tasks
Source: Data Brief. 2022 Apr 27;42:108211. doi: 10.1016/j.dib.2022.108211 (PMC9079685; doi:10.1016/j.dib.2022.108211)
Supplement: Supplementary Data S1 — Supplementary Raw Research Data. This is open data under the CC BY license http://creativecommons.org/licenses/by/4.0/ [file mmc1.pdf]

Supplementary Material

Table 1: Examples from SEOSS-Queries orchestrated of natural language utterances, their category, and the source they were motivated from.

NL utterance	category	motivated by
Return the issue ids of issues of type Bug	development	[1]
Return the issue id, status, resolution, summary and description of issues of type 'Bug' and priority 'Major'	development	[2]
Return the issue id, type and priority of issues with status 'Open' and sort them by type and priority	development	[2]
Return all commit hashes and their corresponding commit messages	development	[3]
What issue type is most frequently mentioned in the database	development	[3]
Count the issue ids of issues that are of status "Open" grouping them by assignee and then give me the assignee and the count of the issue ids of the assignee with most issues assigned to him/her	development	[3]
Return the status type and number of occurences of each status	development	[3]
Return issue pairs where the issue link is 'Blocker'	development	[3]
Give me a list of issues which have a link with the priority "Blocker" or priority "Critical"	development	[3]
What are the emails of the authors of the commits that belong to the issue with id PIG-3592	development	[3]
What fields were changed in fix version 0.12.1	development	[3]
What is the file path of files that were changed in version 0.12.1	development	[3]
List me the number of issues where the resolved date is before 2013-10-27	development	[4]
List all distinct resolutions	-	authors
Return issue fix version and count the issue ids in each version	-	authors
How many issues per updated date were updated	-	authors
On which date were most issues resolved	-	authors
Count all distinct resolutions	research	[5]
Count all distinct assignee usernames	research	[5]
Count number of comments based on the username of commenters	research	[5]

Count number of comments based on display name used in comments. Show the display name and count	research	[5]
Return the component and the occurrence of each component in issues	research	[5]
Give a list with the display name and number of messages left by commenters on issue with id PIG-3592	research	[6]
List commit hashes of change sets made between the created date and resolved date of issue id PIG-3592	research	[7]
How many issues of type bug and type improvement exist in the Apache PIG project	research	[7]
List the issue types of linked source and target issues and their count where the name of the link between them is set to cloners	research	[8]
Return all issues except for those that have their status not set to ”In Progress”	development	[1]
Find all issues which either have the status ”Closed”, ”Resolved” or ”Patch Available”	development	[1]
Return the issue id, type, description of issues that have component ”impl”	development	[4]
Return the issue ids of issues which updated date is between 2017-03-22 and 2017-03-22	development	[1]
Return all information about issue id PIG-3599	development	[1]
Return issues that have assignee set to Daniel Ward and status set to ’Open’	development	[1]
Return issues which status is set to ”In Progress”	development	[1]
Select issues where the reporter is John Stark and the type is Bug	development	[1]
Select issues where the priority is either set to Blocker or Critical	development	[1]
Select issues where the updated date is after ’2017-03-28’	development	[9]
Find issues which assignee is set to Daniel Ward	development	[4]
Search for issues that have attachments	development	[4]
Return the issue id and summary of issues of type ’Bug’	development	[2]
Return the issue id and description of issues of type ’Bug’	development	[2]
Search for issues that belong to grunt component and parser component	development	[4]
What is the status of the issue with id PIG-3599	development	[10]
Return the issue id and priority of issues of type ’Bug’	development	[2]
What fields were changed in version 0.12.1 and by whom. Give me the username of the person responsible for the changes	development	[11]
Return the issue id, type, summary and description of issues that have status ’Open’ and priority ’Major’	development	[2]

Return a list with distinct assignees working on issues of type ’Bug’	development	[11]
Return the author of a commit and number of occurences of each author	development	[3]
Who is the assignee working on issue with the id PIG-3599	development	[3]
How many issues have type ’Improvement’	development	[3]
How many issues are of priority blocker or priority critical	development	[3]
Show me in descending order the top 5 file paths of files to which code changes were made and include the count of commit hashes that belong to each file path	development	[3]
Give me the assignees that are working on only one issue id	development	[3]
List the assignees who were assigned issues of status ”Open” and the number of their assigned issue ids	development	[3]
Return the resolution type and number of occurences of each resolution	development	[3]
Return source issue id and target issues id of issues where the issue link name equals ’Duplicate’	development	[3]
How many issues have status ’Open’	development	[3]
List the assignees and count the issues which status has been set to ”Resolved”	development	[3]
List me display name and message of each person who made a comment on the issue with issue id ”PIG-3599”	development	[3]
How many assignees are working on component impl	development	[3]
Who of the distinct assignees in the database are working on issues in which the component is equal to ”impl”	development	[3]
Which reporter reported what issue ids. Order by reporter	development	[3]
List me the issue ids and assignee of issues where the resolved date of the issues is between 2014-09-08 and 2014-09-12	development	[4]
Find everything about issues that have a created date before ”2014-09-12”	development	[4]
List me all issues which created date was 2017-03-28	development	[4]
Return the issue ids of issues which have resolved date 2013-10-23	development	[4]
Give me a list of all assignees that have resolved issues between the resolved dates ’2015-03-01’ and ’2015-03-31’, limiting the number of issue ids they resolved to three or more	development	[4]
List me issues where the created date is after 2017-05-31	development	[4]
What were the issue ids of issues with resolved date from 2017-10-01 to 2017-10-31	development	[4]

What issues have a created date between '2017-01-01' and '2017-03-31'. Return their issue ids	development	[4]
Return the issue id, type and priority of issues with status 'Open' that have a created date between 2013-11-16 and 2013-11-28. Sort them by type and priority	development	[4]
Return the issue id and status of issues of type 'Bug'	development	[4]
Return the issue id and resolution of issues of type 'Bug'	development	[4]
What is the issue type of issue id PIG-3599	development	[4]
What is the resolved date of the issue with id PIG-3599	development	[4]
Return all information about issues stored in the DB	-	authors
List all distinct priorities	-	authors
List all distinct statuses	-	authors
List all commit hashes	-	authors
Return all distinct e-mails	-	authors
List all distinct components	-	authors
In which fix version were most issues fixed	-	authors
Return the status of the issue with the latest created date	-	authors
In which fix version was PIG-3599 fixed	-	authors
How many issues per date were created	-	authors
How many issues per resolved date were resolved	-	authors
How many commits per date were submitted	-	authors
Return the resolved date and issue ids of issues that Daniel Ward resolved and sort them by resolved date in descending order	-	authors
How many issues did the assignee John Stark resolved? List only those with resolved dates from 2014-10-01 to 2014-10-31	-	authors
Count the issues which have created date between '2015-02-01' and '2015-04-30'	-	authors
On which date most issues were created	-	authors
Count all rows containing issue information in the database	research	[5]
Count all unique issue types	research	[5]
Return the issue type and number of occurences of each type	research	[5]
Count all distinct priorities	research	[5]
Return the priority and number of occurences of each priority	research	[5]
Count all distinct statuses	research	[5]
Count all distinct assignees	research	[5]
Return the different assignee and number of occurences of each assignee	research	[5]
Return the assignee username and number of occurences of each assignee username	research	[5]

Count all distinct reporters	research	[5]
Return the reporter and number of occurences of each reporter	research	[5]
Count all distinct reporter usernames	research	[5]
Return the reporter username and number of occurences of each reporter username	research	[5]
Count all commits	research	[5]
Count all distinct authors	research	[5]
Return all distinct usernames that comment on issues	research	[5]
Count all distinct components	research	[5]
Count the number of distinct issue link names	research	[5]
Return the name of issue links and their occurrence	research	[8]
Count the issues of type 'Bug'	research	[5]
List all distinct assignees	research	[12]
List all distinct assignees usernames	research	[12]
List all distinct reporters	research	[6]
List all distinct reporter usernames	research	[6]
List all distinct authors of commits	research	[12]
Return for each issue the issue ids, created date, resolved date, as well as commit hashes associated to an issue and their committed dates	research	[12]
List issues linked to change sets	research	[12]
How many issues are not linked to change sets	research	[12]
How many issues have more than one commit hash	research	[12]
How many issues have only one commit hash linked to them	research	[12]
List the username of commenters, as well as the number of distinct issues ids associated with each username	research	[6]
List the issue id of issues that have comments and include the count of unique usernames of developmentelopers who made a comment on each issue from the list	research	[6]
Return the issue id and number of messages left on commented issues	research	[6]
List issue id, summary and description of issues	research	[13]
How many file paths are linked to issue id PIG-3599	research	[7]
Return the minimum number of file paths of modified files which can be associated with issue ids of issues of type 'Bug'	research	[7]
How many linked issues does the DB contain where the source issue is of type bug while the target issue is of type improvement	research	[7]
List the source and target issue ids of issues that are linked where the source issue is of type Improvement while the target issue is of type Bug	research	[7]

Return the distinct names of issue links	research	[8]
List the issue types of linked source and target issues where the name of the link between them is set to cloners	research	[8]
List all distinct issue types	research	[14]
Return issues of type 'Bug'	research	[15]
Return the maximum number of file paths of modified files which can be associated with issue ids of issues of type 'Improvement'	research	[7]
What issues have no assignee	development	[1]
Return the issue id and description of all issues except those without description	development	[1]
Search for issues that have no attachments	development	[4]
Return all issues that have no description	development	[1]
What issues are unassigned and have not been updated since '2013-09-08'	development	[1]
Is the issue link name of issue pairs with either source or target id PIG-3543 equal to Duplicate	development	[2]
Return the issue which is linked to either a source or target issue id PIG-3543 and which issue link name is 'Duplicate'	development	[2]
Return the source issue id, target issue id , the descriptions of the source and target issues which link name is set to "Duplicate"	development	[2]
Return the source issue id, target issue id, the descriptions of the source and target issues and the commit hashes and commit messages of the source and target issues which link name is set to "Reference"	development	[2]
Return the components of the source and target issue pairs that are linked and the link name equals "Blocker"	development	[2]
Return everything about issues where the resolved date is not empty and the assignee is Daniel Ward	development	[11]
Return the assignee and count the issue ids that have satus null and the assignee is not empty	development	[3]
Who of the assignees are working on more than one issue that is set to either status "Open" or status "In Progress"	development	[3]
Count the assignees that are working on a single issue id	development	[3]
Who is the assignee with most issues ids of status "Resolved"	development	[3]
How many issues don't have description	research	[5]
Return the issue ids of issues created yesterday	development	[4]
Find everything about issues with a Fix Version of 0.12.1	development	[4]
List me the issue id, type, description and comments of issues with resolution "Won't fix"	development	[4]

Find everything about issues with a resoltion of "Cannot Reproduce" or "Won't fix"	development	[4]
List the issue id, summary, description of issues and the filenames and mime types of any attachment of issues of type "Bug" that are still unassigned	-	authors
Return the description and file names of attachments of issues id PIG-3599	-	authors
Is issue id PIG-4092 linked to commit hash 84889e7852c98c67b40d12116e3bb6dd311a3363	research	[12]
Average number of comments based on all issues	research	[6]
Maximum number of comments based on the count of comments per issue id	research	[6]
Which issues have comments? Give me everything about the issue	research	[6]
Select issues of type Bug with status "Resolved" or "Closed and resolution "Fixed" or "Done"	research	[13]
In which issues was the file in the file path 'src/org/apache/pig/builtin/ParquetStorer.java' modified	research	[7]
What is the average number of file paths of modified files which can associated with issue ids of issues of type 'Bug'	research	[7]
Show me the source issue id, target issue id, summary and description of the source and target issues that have a link with the name Cloners or Reference	research	[7]
Maximum number of unique usernames of commenters based on all issues	research	[6]
Average number of commenters per issue	research	[6]
List me the assignees assigned to issue id "PIG-4648" and issue id "PIG-4370"	development	[3]

References

[1] Jira blog, ???? URL: https://www.atlassian.com/blog/jira-software/jql-the-most-flexible-way-to-search-jira-14.

[2] A. J. Ko, R. DeLine, G. Venolia, Information needs in collocated software development teams, in: ICSE, IEEE Computer Society, 2007, pp. 344–353.

[3] A. Abdellatif, K. Badran, E. Shihab, Msrbot: Using bots to answer questions from software repositories, Empir. Softw. Eng. 25 (2020) 1834–1863.

[4] Jira software cloud, ???? URL: https://support.atlassian.com/jira-software-cloud/docs/advanced-search-reference-jql-fields/.

[5] A. Bachmann, A. Bernstein, Software process data quality and characteristics: a historical view on open and closed source projects, in: EVOL/IWPSE, ACM, 2009, pp. 119–128.

[6] M. Rath, P. Mäder, Request for comments: conversation patterns in issue tracking systems of open-source projects, in: SAC, ACM, 2020, pp. 1414–1417.

[7] M. Rath, D. Lo, P. Mäder, Analyzing requirements and traceability information to improve bug localization, in: MSR, ACM, 2018, pp. 442–453.

[8] M. T. Tomova, M. Rath, P. Mäder, Use of trace link types in issue tracking systems, in: ICSE (Companion Volume), ACM, 2018, pp. 181–182.

[9] Jira guides developers, ???? URL: https://www.atlassian.com/software/jira/guides/expand-jira/jql#visualize-results.

[10] S. Breu, R. Premraj, J. Sillito, T. Zimmermann, Information needs in bug reports: improving cooperation between developers and users, in: CSCW, ACM, 2010, pp. 301–310.

[11] T. Fritz, G. C. Murphy, Using information fragments to answer the questions developers ask, in: ICSE (1), ACM, 2010, pp. 175–184.

[12] M. Rath, J. Rendall, J. L. C. Guo, J. Cleland-Huang, P. Mäder, Traceability in the wild: Automatically augmenting incomplete trace links, in: SE/SWM, volume P-292 of *LNI*, GI, 2019, p. 63.

[13] M. Rath, P. Mäder, Influence of structured information in bug report descriptions on ir-based bug localization, in: SEAA, IEEE Computer Society, 2018, pp. 26–32.

[14] T. Merten, B. Mager, P. Hübner, T. Quirchmayr, B. Paech, S. Bürsner, Requirements communication in issue tracking systems in four open-source projects, in: REFSQ Workshops, volume 1342 of *CEUR Workshop Proceedings*, CEUR-WS.org, 2015, pp. 114–125.

[15] T. Merten, M. Falis, P. Hübner, T. Quirchmayr, S. Bürsner, B. Paech, Software feature request detection in issue tracking systems, in: RE, IEEE Computer Society, 2016, pp. 166–175.
